# Digital data for Quick Response (QR) codes of thermophiles to identify and compare the bacterial species isolated from Unkeshwar hot springs (India)

**DOI:** 10.1016/j.dib.2015.11.035

**Published:** 2015-11-24

**Authors:** Bhagwan N. Rekadwad, Chandrahasya N. Khobragade

**Affiliations:** School of Life Sciences, Swami Ramanand Teerth Marathwada University, Nanded, Maharashtra 431606, India

**Keywords:** DNA bank, DNA signatures, Microbial diversity informatics, Thermal springs

## Abstract

16S rRNA sequences of morphologically and biochemically identified 21 thermophilic bacteria isolated from Unkeshwar hot springs (19°85′N and 78°25′E), Dist. Nanded (India) has been deposited in NCBI repository. The 16S rRNA gene sequences were used to generate QR codes for sequences (FASTA format and full Gene Bank information). Diversity among the isolates is compared with known isolates and evaluated using CGR, FCGR and PCA i.e. visual comparison and evaluation respectively. Considerable biodiversity was observed among the identified bacteria isolated from Unkeshwar hot springs. The hyperlinked QR codes, CGR, FCGR and PCA of all the isolates are made available to the users on a portal https://sites.google.com/site/bhagwanrekadwad/.

**Specifications Table**

**Table:** 

Subject area	*Biology*
More specific subject area	*Microbial diversity Informatics*
Type of data	*Text file, sequences, table, Quick Response Codes (QR Codes), Chaos Game representation (CGR) and Chaos Game Representation of Frequencies (FCGR), neighbor joining(NJ) plot and Principal Component Analysis (PCA) images*
How data was acquired	*Amplified Biosystems Model 3730 XI (96 capillary) DNA sequencer*
Data format	*Raw and analyzed*
Experimental factors	*DNA fragments were obtained using slightly modified Phenol-Chloroform method.*
Experimental features	*Genomic DNA fragmented and then sequenced using Sanger*′*s dideoxy DNA sequencing method using Amplified Biosystems DNA sequencer. 16SrRNA gene sequences were used to create QR codes using DNA BarID software.*
Data source location	*Unkeshwar* (19°85′N and 78°25′E)*, School of Life Sciences, Swami Ramanand Teerth Marathwada University, Nanded, India* (19°6′N and 78°17′E).
Data accessibility	1. *Raw data is available through NCBI׳s BioSample database* (www.ncbi.nlm.nih.gov/nuccore)*. BioSample IDs include JN392966-JN392971, KC120909-KC120919, KM KM998072-KM998074 and KP053645.* 2. *Data is with this article made available to users* a. *Each isolates have two hyperlinked QR codes, CGR, FCGR.* b. *Names of isolates, Accession Numbers, QR codes, CG and PCR, FCGR and PCA of isolates made available on internet on website created by us* https://sites.google.com/site/bhagwanrekadwad/

**Value of the data**

• Microbial community isolated from Unkeshwar hot spring has enormous biotechnological applications. Generated digital information is a limelight for identification and comparison of newly isolated microorganisms.
• This digitization of 16S RNA sequences of thermophiles were carried out first time by us from Unkeshwar hot spring and made available to users.
• This generated digital information provides a baseline to any researchers by reducing time and cost on identification and comparison of bacterial diversity in hot springs.
• The DNA sequence data digitization is a standard, fast and reliable tool for identification of microorganisms up to species level using short DNA sequences.

## 1 Experimental design, materials and methods

The Sanger׳s dideoxy method was adopted for DNA sequencing. 16S rRNA gene sequence analysis was carried out to confirm the identity of bacteria using morphological and biochemical tests. The bacterial cultures were enriched in a nutrient agar medium and the DNA was extracted using a phenol–chloroform method with slight modification. The method was modified as follow. About 2 mL of cell pellet from each enrichment culture of isolate was suspended in extraction buffer containing (100 mM Tris–HCl, pH 8.0, 100 mM Na_2_EDTA (pH 8.0) and Proteinase K (Nitrogen, USA) at the final concentration of 100 mg/mL. The resulting mixture was incubated at 55 °C for 2 h with continuous shaking. To this 0.5 M NaCl was added and incubated at 72 °C for 30 min. Subsequently, DNA was extracted by phenol:chloroform:isoamyl alcohol (1:1:1). It was washed twice with 70% ethanol and dissolved in Tris-EDTA buffer. The DNA was analyzed by electrophoresis in a 0.8% agarose gel stained with ethidium bromide and visualized under UV trans-illuminator. The 16S rDNA of the enriched strains were amplified with two different pair of eubacteria specific primers (forward primer 530 F: 5′ GTGCCAGCAGCCGCGG 3′ and reverse primer 1392 R: 5′ACGGGCGGTGTGTAC 3′ and forward primer Bac 8F: 5′ AGAGTTTGATCCTGGCTCAG 3′ and reverse primer 1492 R: 5′ GGTTACCTTGTTACGACTT 3′). The PCR conditions used were an initial denaturation at 94 °C for two minutes, followed by 35 cycles of denaturation at 95 °C for one minute, annealing at 55 °C for one minute and extension at 72 °C for one minute. Finally, extension was given at 72 °C for 10 min. The PCR products were electrophoresed in 1% (w/v) agarose gel containing ethidium bromide (1 µg mL^−1^) so as to get fragments of DNA. The resulting products were purified and directly sequenced on the Amplified Biosystem Model 3730 XI (96 capillaries) DNA sequencer (Amplified Biosystems, Inc., Foster City, Calif, USA). The sequences of bacterial isolates were determined through a BLAST search. Nucleotide sequences were aligned using the software MEGA 6. The phylogenetic tree was constructed by the neighbor-joining method using a distance Matrix from the alignment. Tree files were generated by PHYLIP and viewed by TREEVIEW program. Bootstrap analysis was also carried out to know the evolutionary history of bacteria [1], [2], [3], [4].

## 2 Data

The DNA QR codes of identified bacterial species were generated using DNA BarID downloaded from NEERI-CSIR, Nagpur website. The generated QR codes for the species (Table 1) of bacteria have unique QR codes (Table 2) which do not resembles with any other species or strains in any database. Using these QR codes any smart user can scan QR code and read more information on bacterial species. This information is useful to identify and compare the QR-coded isolates or sequences isolated from hot spring environment/extremes.

**Fig. 1:**
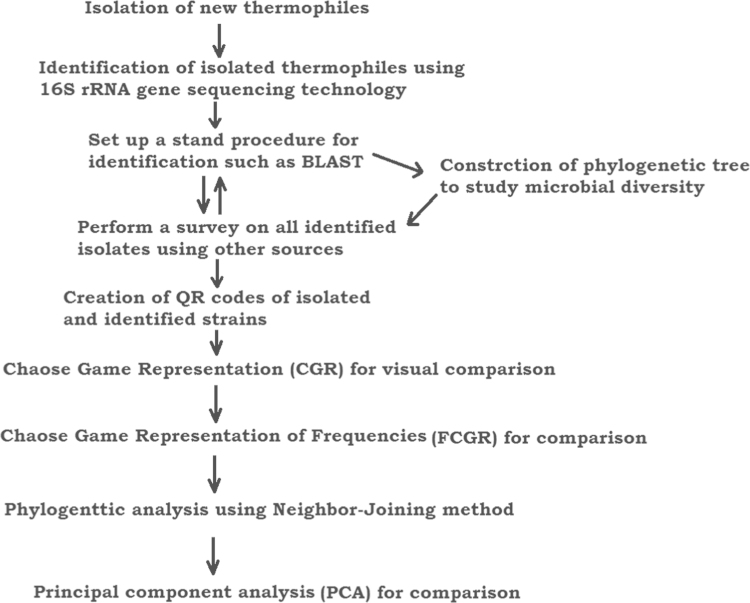
Diagram shows constructive flow chart to assess microbial diversity and its digitization.

**Fig. 2:**
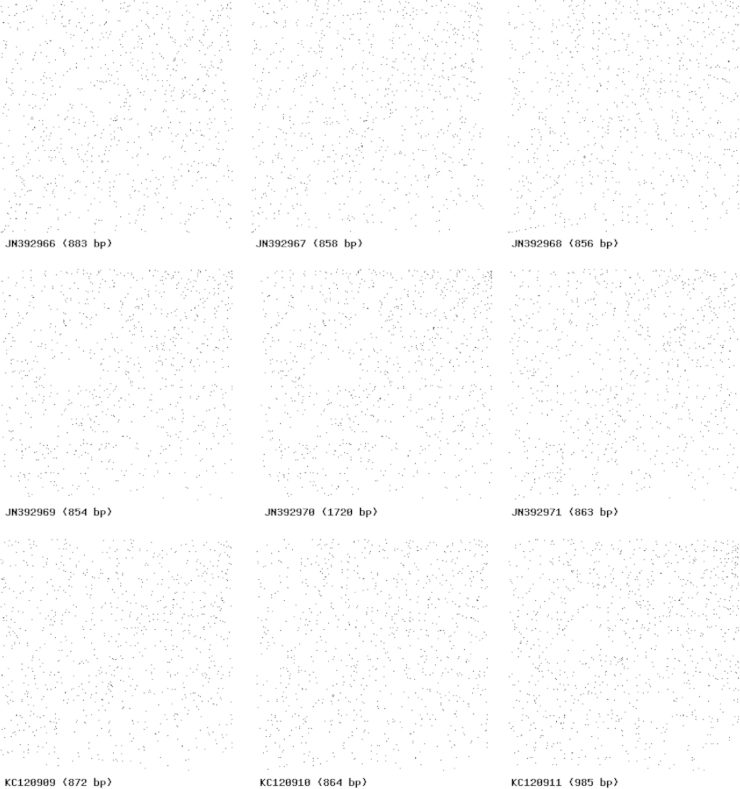

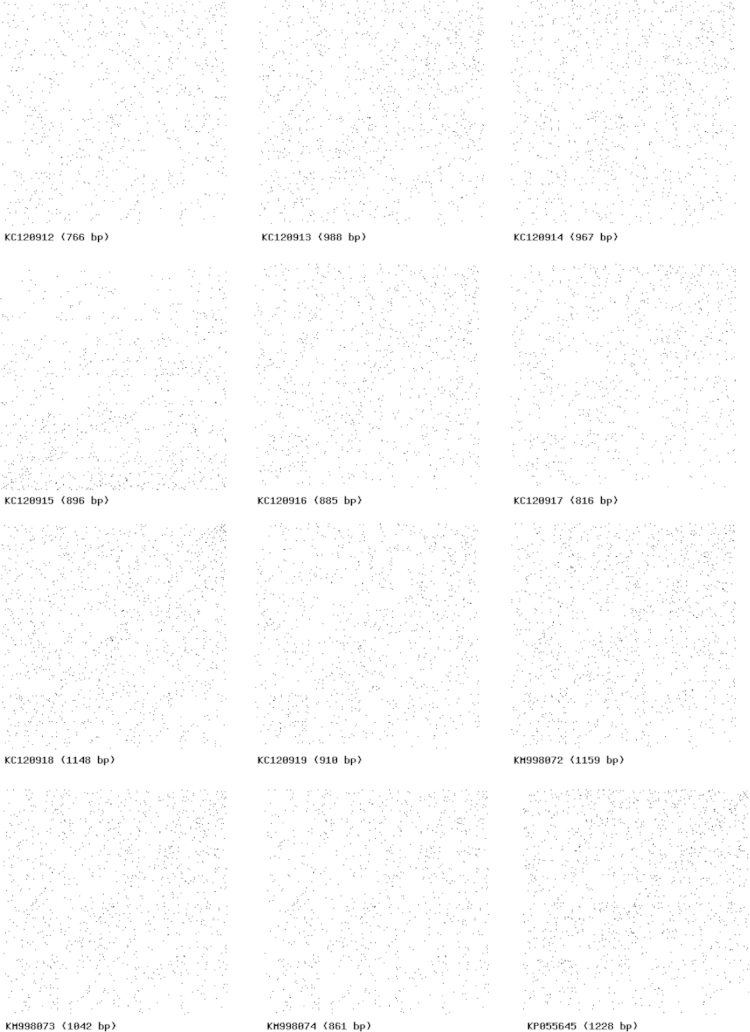
Chaos Game representation (CGR) codes of isolates showing difference in composition of DNA base sequences.

**Fig. 3:**
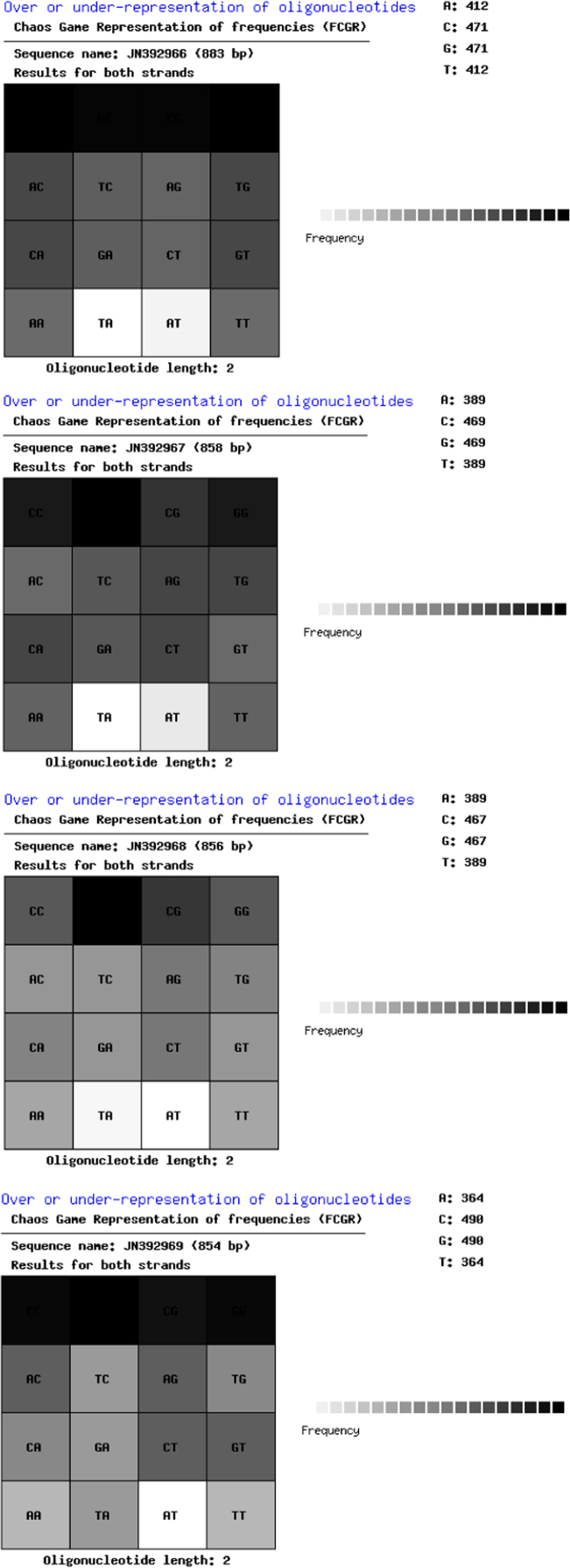

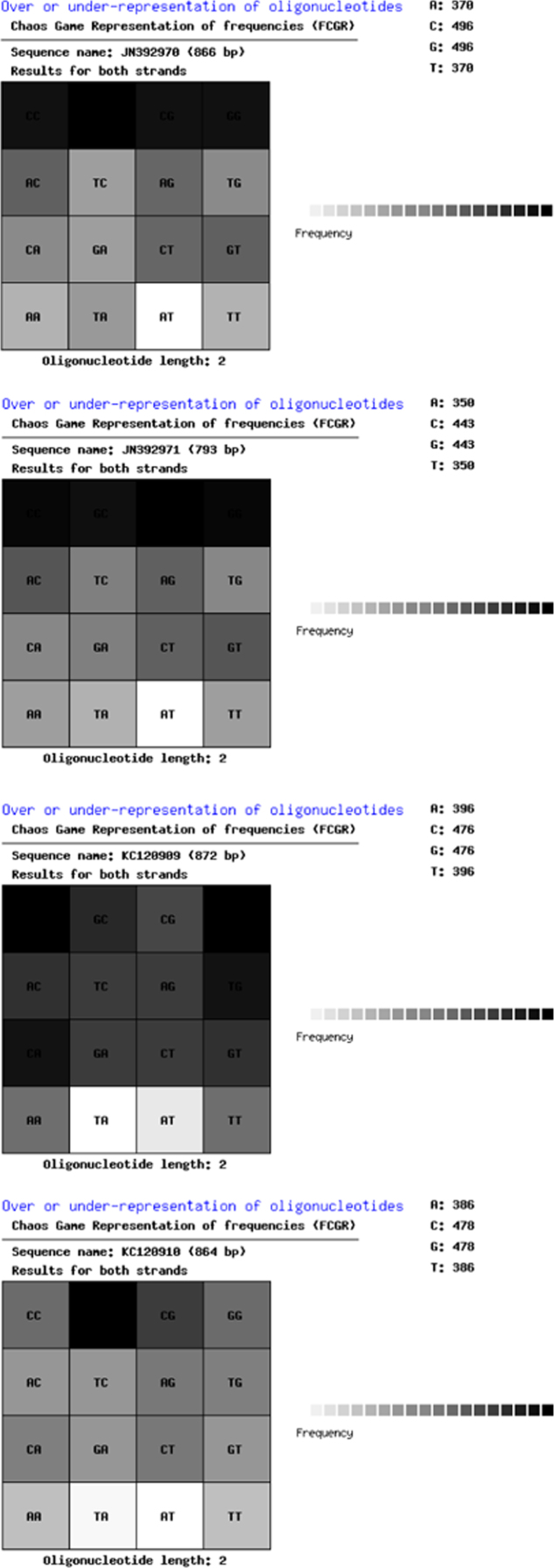

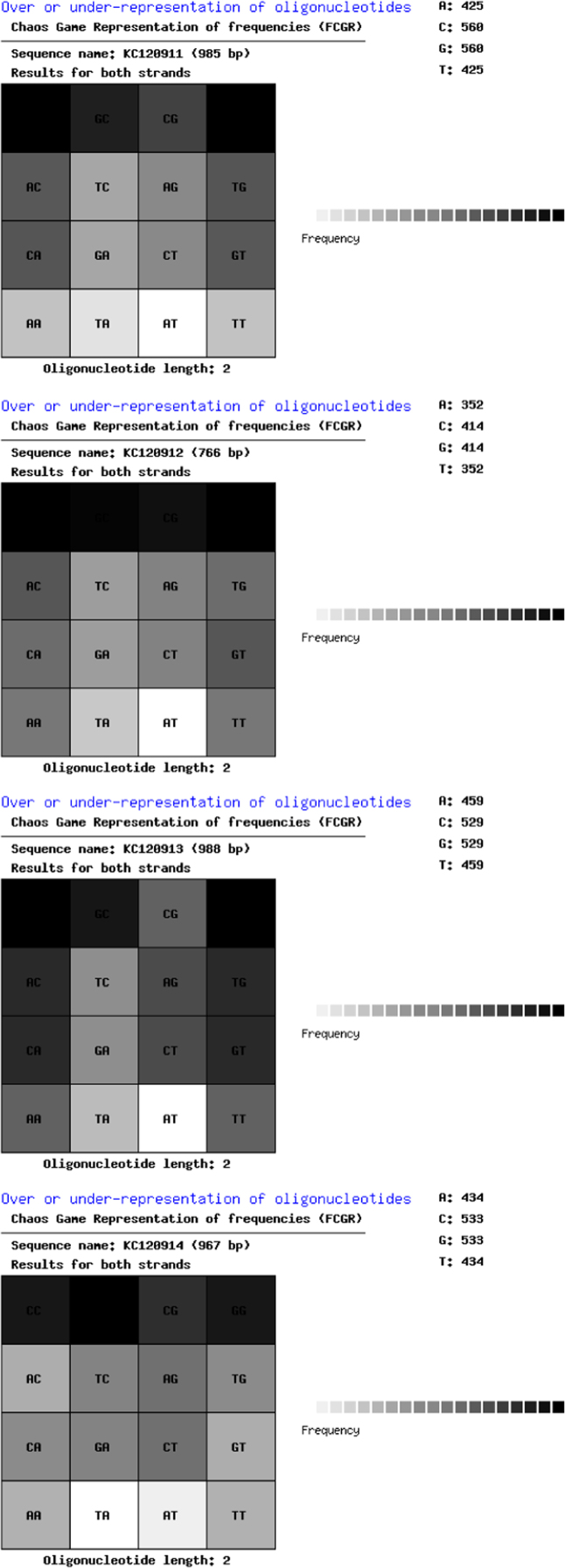

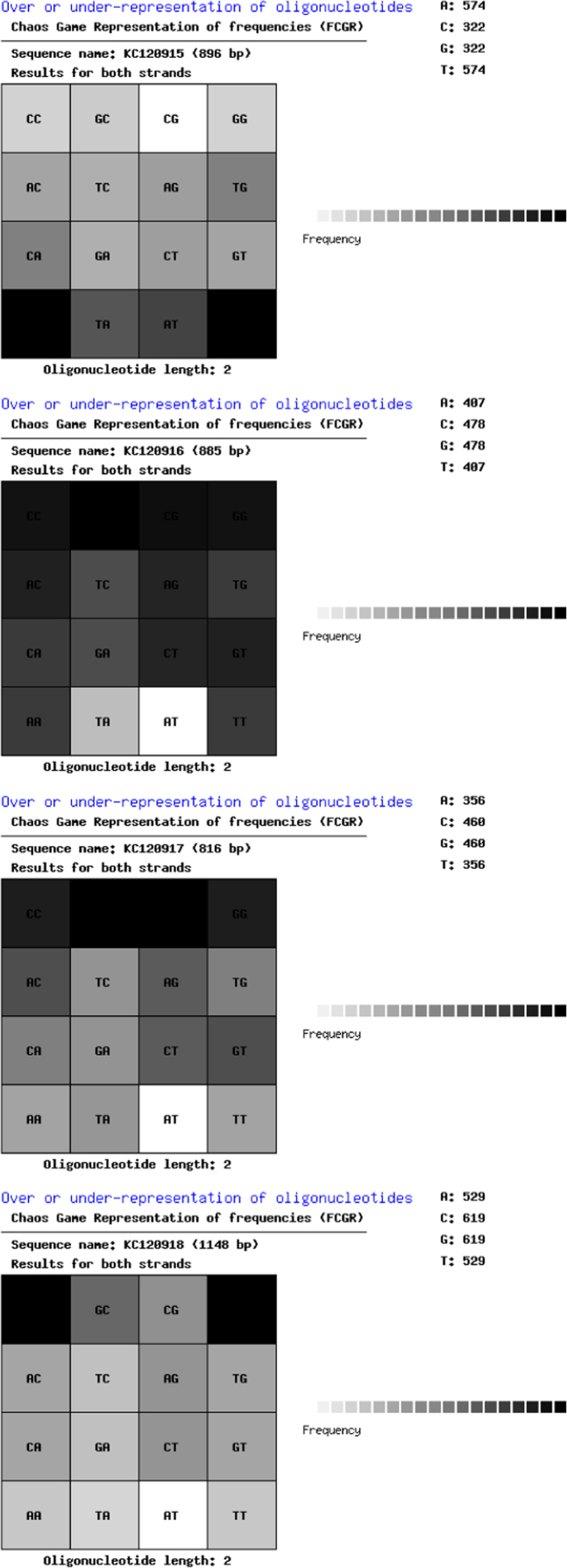

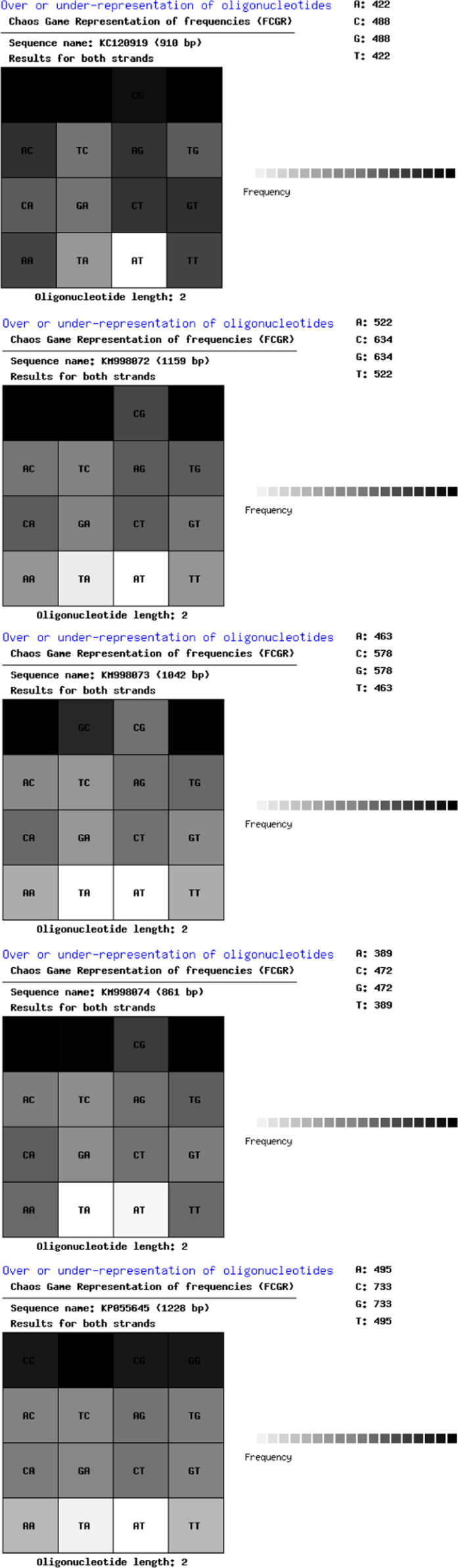
Chaos Game Representation of frequencies (FCGR) of isolates.

The generated data were compared with other visual techniques such as CGR and FCGR. The phylogenetic tree was constructed using MEGA6 and PCA for comparative analysis (Fig. 1, Fig. 2, Fig. 3).

## 3 Digitization and microbial diversity informatics

**Fig. 4:**
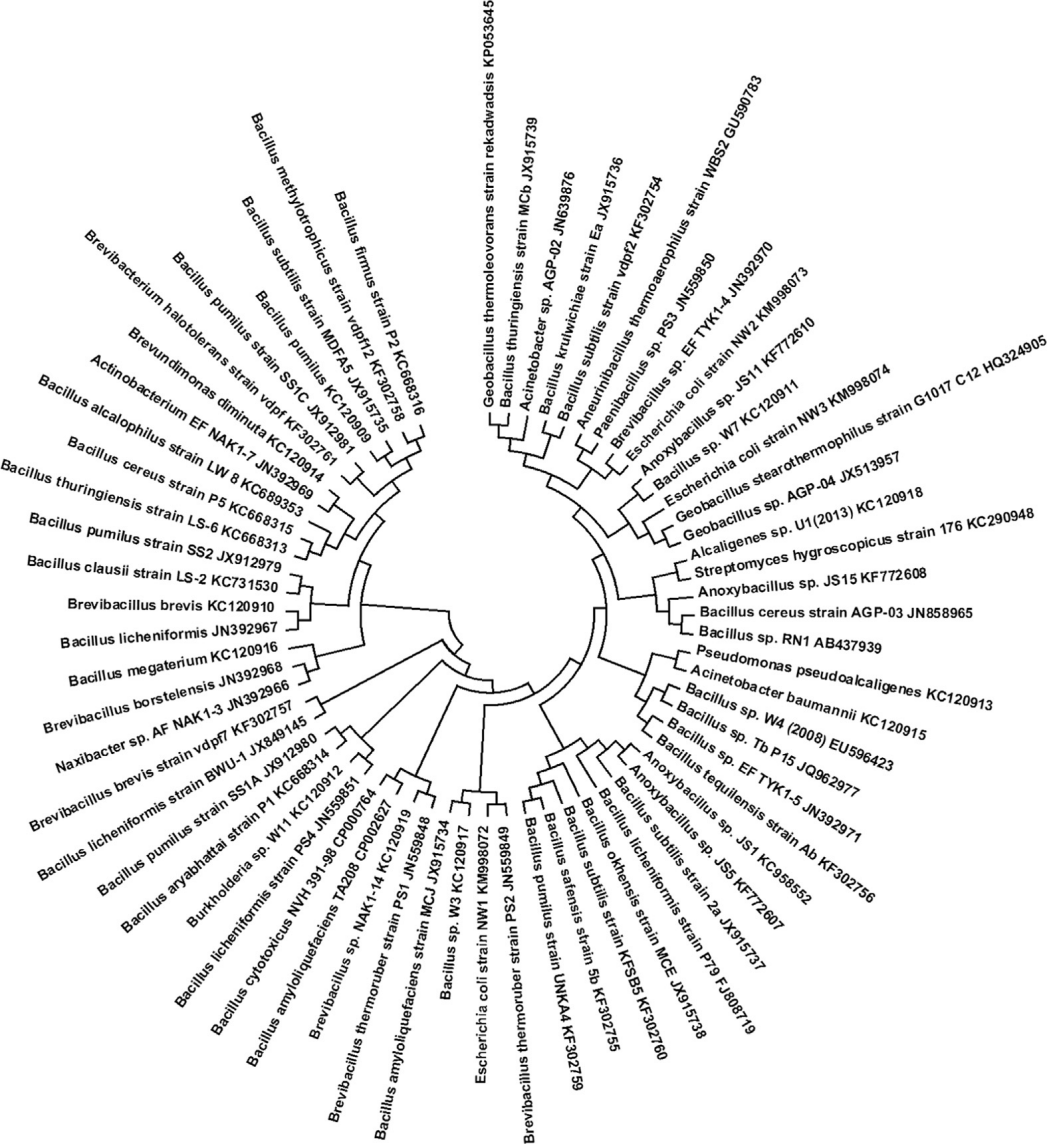
Evolutionary relationships of taxa (JN392966-JN392971, KC120909-KC120919, KM998072-KM998074 and KP053645 with other species isolated from hot springs). The evolutionary history was inferred using the Neighbor-Joining method [6]. The bootstrap consensus tree inferred from 1000 replicates is taken to represent the evolutionary history of the taxa analyzed [7]. Branches corresponding to partitions reproduced in less than 50% bootstrap replicates are collapsed. The evolutionary distances were computed using the Maximum Composite Likelihood method [8] and are in the units of the number of base substitutions per site. The analysis involved 65 nucleotide sequences. All positions containing gaps and missing data were eliminated. There were a total of 591 positions in the final dataset. Evolutionary analyses were conducted in MEGA6 [9].

**Fig. 5:**
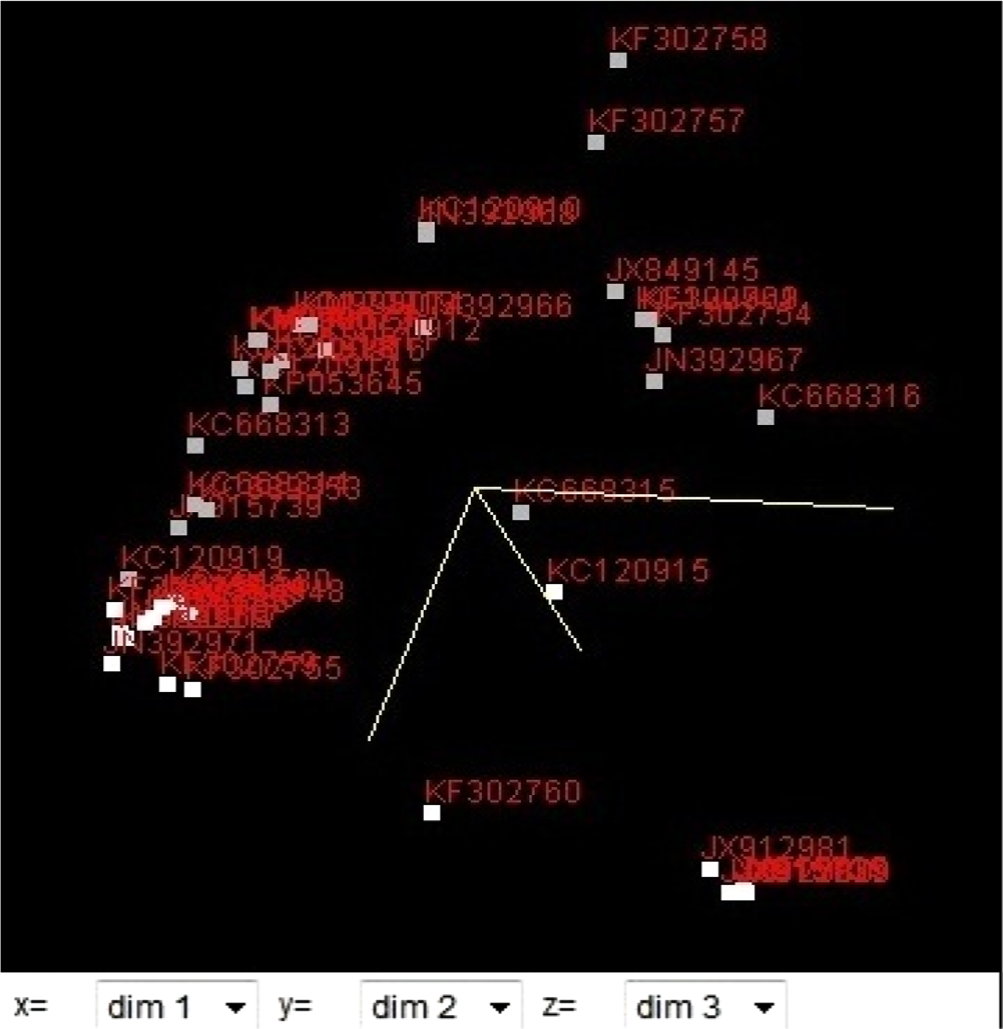
Principal component analysis (PCA) of isolates.

QR codes for 16S rRNA gene sequences in FASTA format and for full Gene Bank information was generated using DNA BarID software developed by Purohit et al. [5]. The diversity of microorganisms isolated from various hot springs including Unkeshwar, District Nanded, India (19°85′N and 78°25′E) were observed and compared using phylogenetic tree and PCA (Fig. 4, Fig. 5).

## 4 QR codes hyper links

The QR codes were hyperlinked using Microsoft word processor software. The QR codes of 21 identified bacteria available to any user on a portal https://sites.google.com/site/bhagwanrekadwad/.

## 5 Bacterial sequences

The FASTA format sequences and Gene Bank (full) information of 16S rRNA sequences of 21 isolated bacteria identified by us are taken for digitization. 16S rRNA sequences of identified strains submitted to NCBI repository with accession numbers JN392966-JN392971, KC120909-KC120919, KM998072-KM998074 and KP053645. Using 16S rRNA sequences, the generated QR codes, CGR, FCGR and PCA were made available to any user on website https://sites.google.com/site/bhagwanrekadwad/.

## Figures and Tables

**Table 1:** Names and accession numbers of QR coded isolates isolated and identified Unkeshwar hot springs.

**Species**	**Accession numbers**
*Naxibacter* sp. AF_NAK1-3	JN392966
*Bacillus licheniformis*	JN392967
*Brevibacillus borstelensis*	JN392968
*Actinobacterium* EF_NAK1-7	JN392969
*Brevibacillus* sp. EF_TYK1-4	JN392970
*Bacillus* sp. EF_TYK1-5	JN392971
*Bacillus pumilus*	KC120909
*Brevibacillus brevis*	KC120910
*Bacillus* sp. W7	KC120911
*Burkholderia* sp. W11	KC120912
*Pseudomonas pseudoalcaligenes*	KC120913
*Brevundimonas diminuta*	KC120914
*Acinetobacter baumannii*	KC120915
*Bacillus megaterium*	KC120916
*Bacillus* sp. W3	KC120917
*Alcaligenes* sp. U1(2013)	KC120918
*Brevibacillus* sp. NAK1-14	KC120919
*Escherichia coli* strain NW1	KM998072
*Escherichia coli* strain NW2	KM998073
*Escherichia coli* strain NW3	KM998074
*Geobacillus thermoleovorans* strain rekadwadsis	KP053645

**Table 2:** QR code generated for FASTA format sequences and Gene Bank (full) information using DNA BarID software.



