# Supplementary figures and images for: Digital data for Quick Response (QR) codes of thermophiles to identify and compare the bacterial species isolated from Unkeshwar hot springs (India)
Source: Data Brief. 2015 Nov 24;6:53–67. doi: 10.1016/j.dib.2015.11.035 (PMC4688402; doi:10.1016/j.dib.2015.11.035)

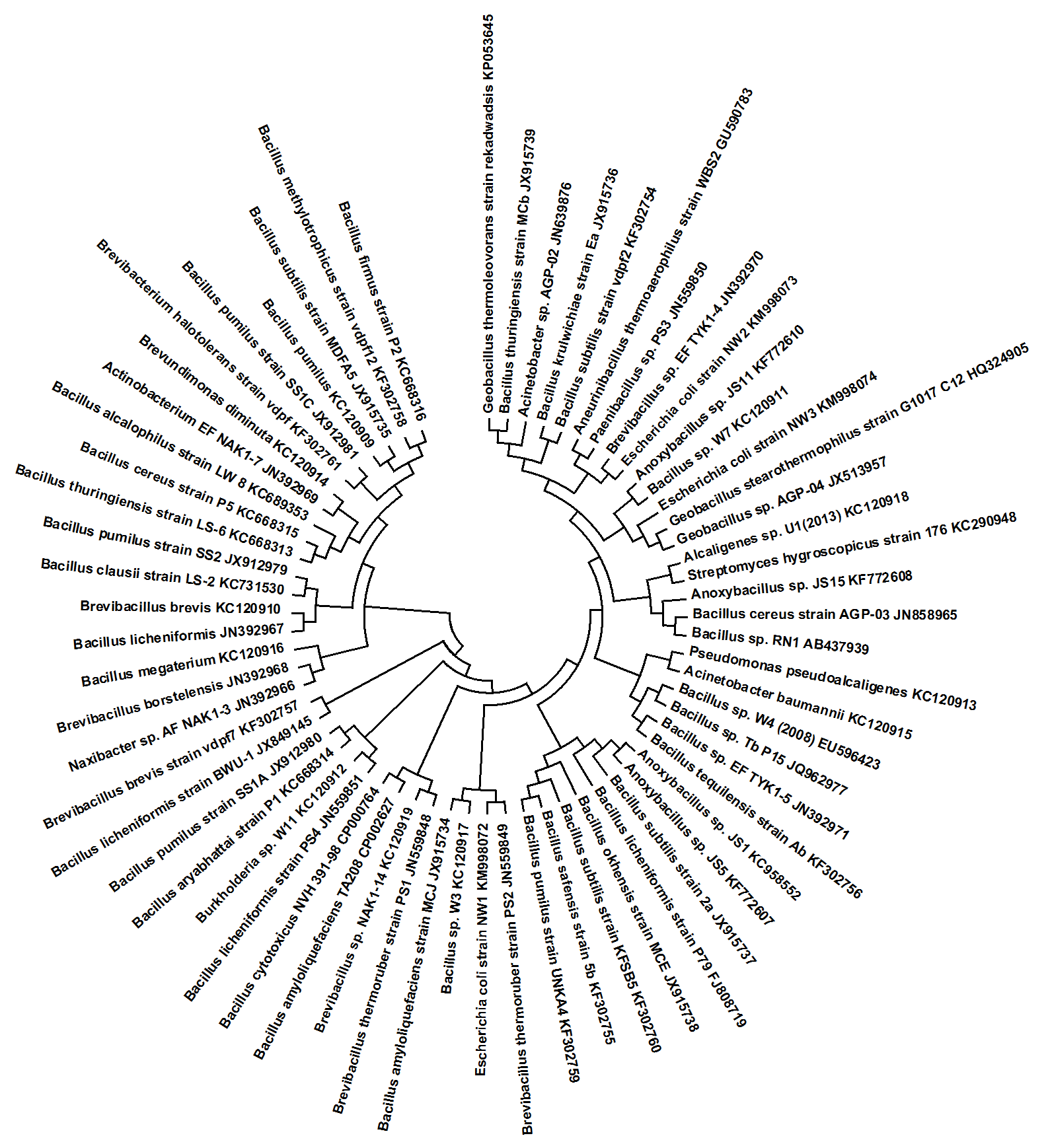

Supplement: Supplementary file 1 — Supplementary material [file mmc1.zip › BARCODE PAPER NJ 11 0 9 15.png]

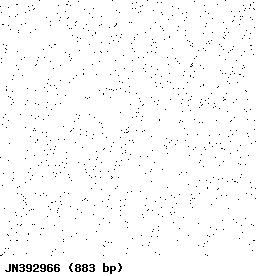

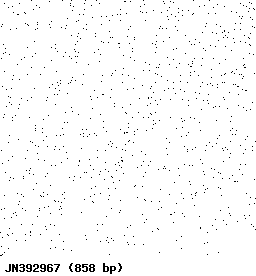

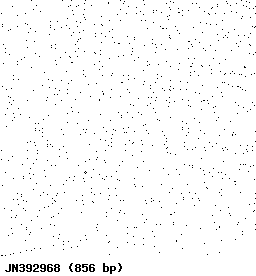


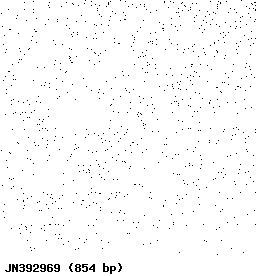

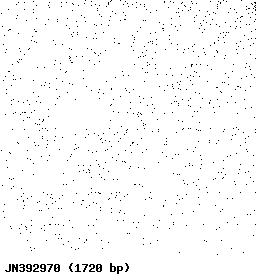

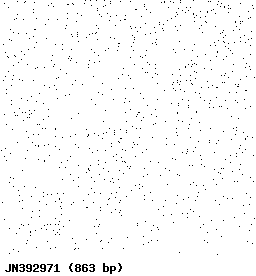


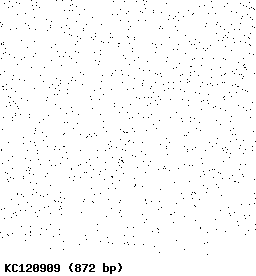

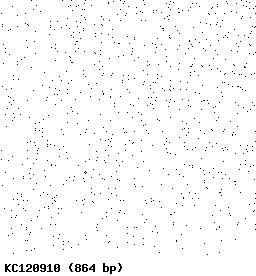

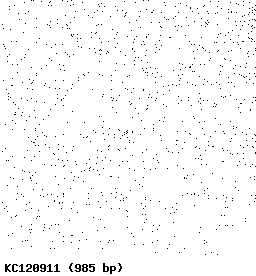


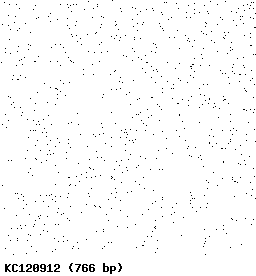

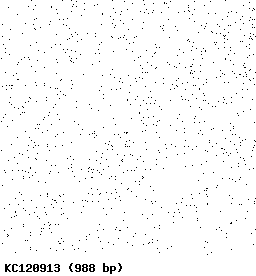

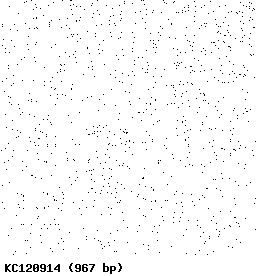


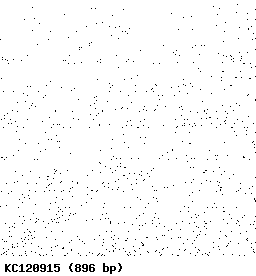

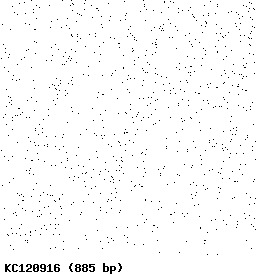

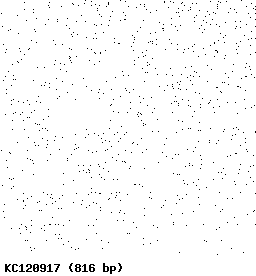


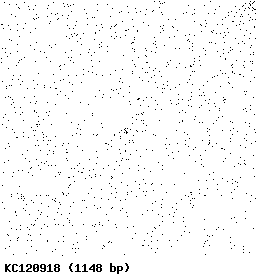

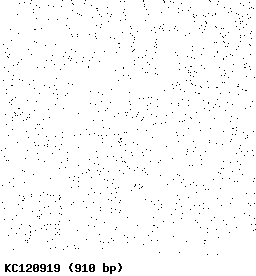

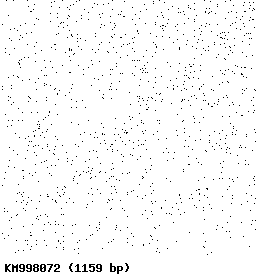


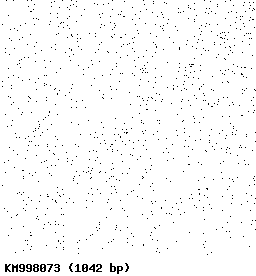

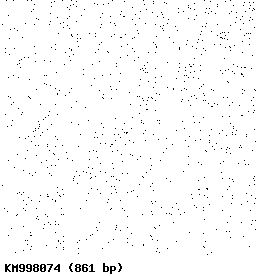

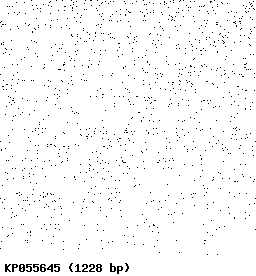


Figure: CGR codes of isolates

Supplement: Supplementary file 2 — Supplementary material [file mmc2.zip › Chaose Game Representation (CGR)/cgr.docx]

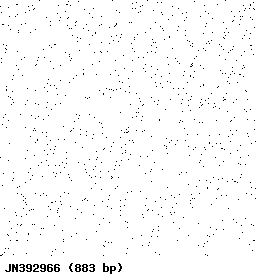

Supplement: Supplementary file 2 — Supplementary material [file mmc2.zip › Chaose Game Representation (CGR)/JN392966.png]

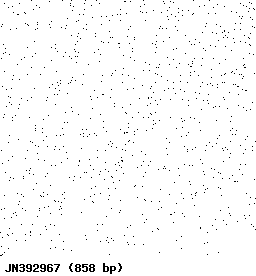

Supplement: Supplementary file 2 — Supplementary material [file mmc2.zip › Chaose Game Representation (CGR)/JN392967.png]

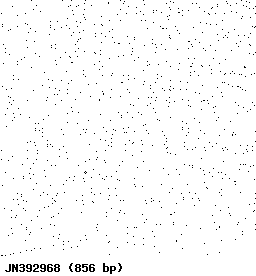

Supplement: Supplementary file 2 — Supplementary material [file mmc2.zip › Chaose Game Representation (CGR)/JN392968.png]

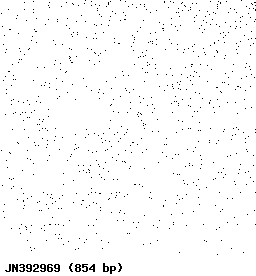

Supplement: Supplementary file 2 — Supplementary material [file mmc2.zip › Chaose Game Representation (CGR)/JN392969.png]

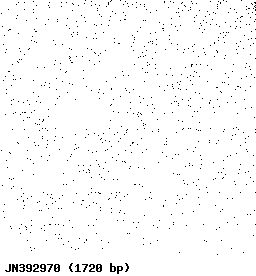

Supplement: Supplementary file 2 — Supplementary material [file mmc2.zip › Chaose Game Representation (CGR)/JN392970.png]

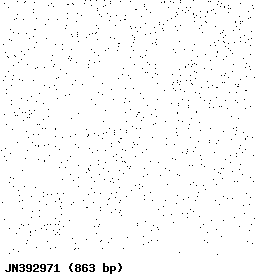

Supplement: Supplementary file 2 — Supplementary material [file mmc2.zip › Chaose Game Representation (CGR)/JN392971.png]

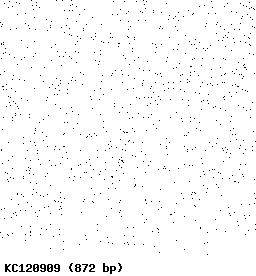

Supplement: Supplementary file 2 — Supplementary material [file mmc2.zip › Chaose Game Representation (CGR)/KC120909.png]

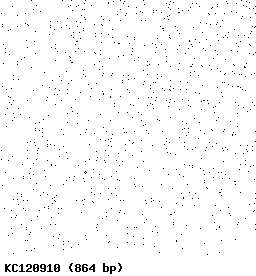

Supplement: Supplementary file 2 — Supplementary material [file mmc2.zip › Chaose Game Representation (CGR)/KC120910.png]

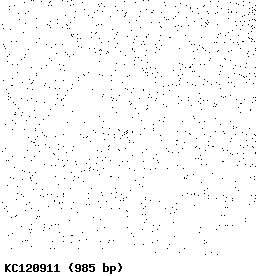

Supplement: Supplementary file 2 — Supplementary material [file mmc2.zip › Chaose Game Representation (CGR)/KC120911.png]

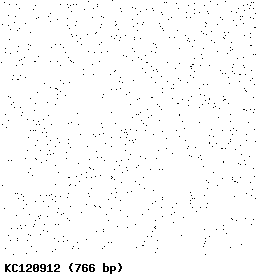

Supplement: Supplementary file 2 — Supplementary material [file mmc2.zip › Chaose Game Representation (CGR)/KC120912.png]

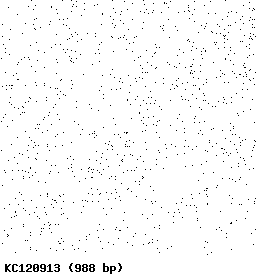

Supplement: Supplementary file 2 — Supplementary material [file mmc2.zip › Chaose Game Representation (CGR)/KC120913.png]

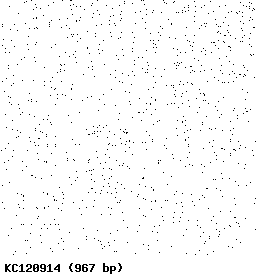

Supplement: Supplementary file 2 — Supplementary material [file mmc2.zip › Chaose Game Representation (CGR)/KC120914.png]

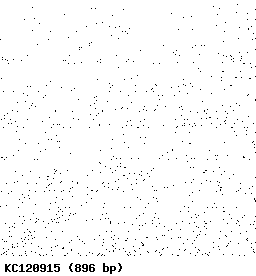

Supplement: Supplementary file 2 — Supplementary material [file mmc2.zip › Chaose Game Representation (CGR)/KC120915.png]

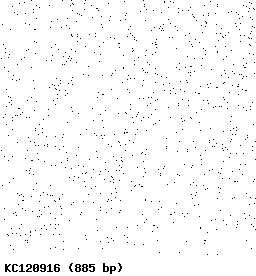

Supplement: Supplementary file 2 — Supplementary material [file mmc2.zip › Chaose Game Representation (CGR)/KC120916.png]

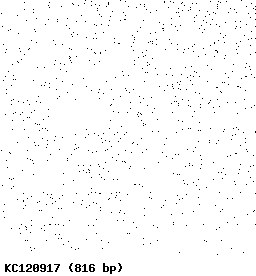

Supplement: Supplementary file 2 — Supplementary material [file mmc2.zip › Chaose Game Representation (CGR)/KC120917.png]

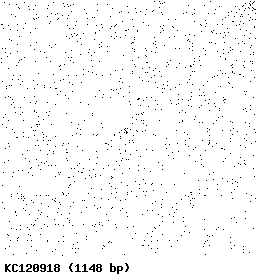

Supplement: Supplementary file 2 — Supplementary material [file mmc2.zip › Chaose Game Representation (CGR)/KC120918.png]

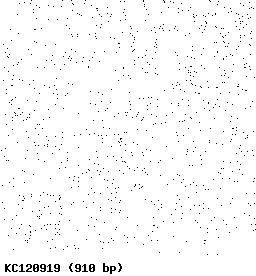

Supplement: Supplementary file 2 — Supplementary material [file mmc2.zip › Chaose Game Representation (CGR)/KC120919.png]

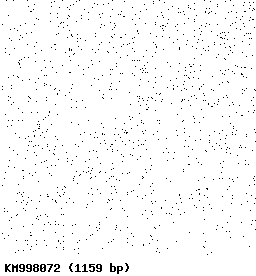

Supplement: Supplementary file 2 — Supplementary material [file mmc2.zip › Chaose Game Representation (CGR)/KM998072.png]

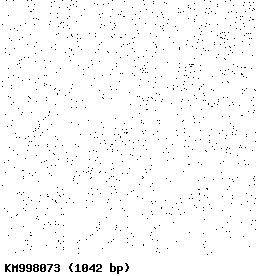

Supplement: Supplementary file 2 — Supplementary material [file mmc2.zip › Chaose Game Representation (CGR)/KM998073.png]

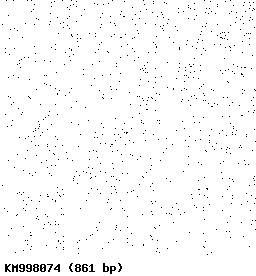

Supplement: Supplementary file 2 — Supplementary material [file mmc2.zip › Chaose Game Representation (CGR)/KM998074.png]

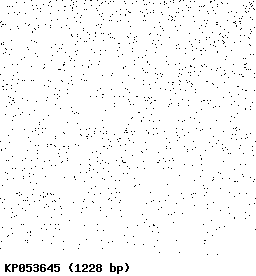

Supplement: Supplementary file 2 — Supplementary material [file mmc2.zip › Chaose Game Representation (CGR)/KP053645.png]

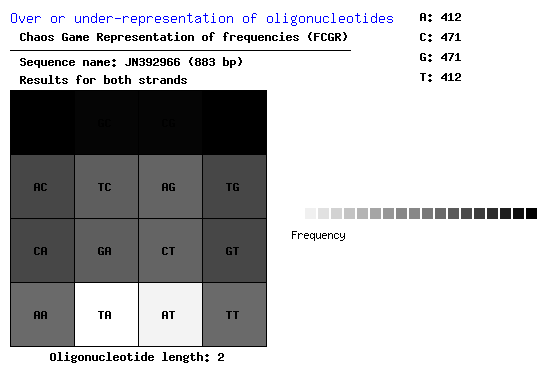


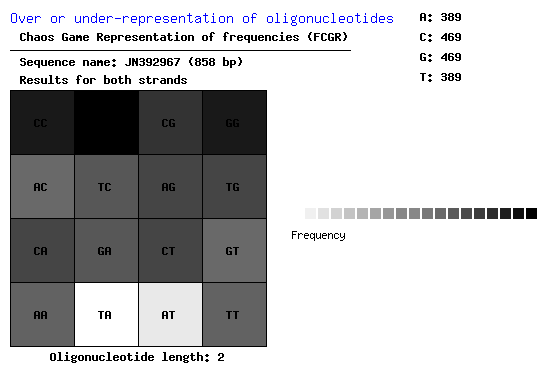


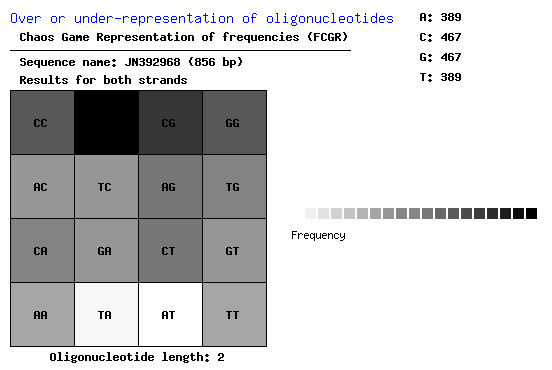


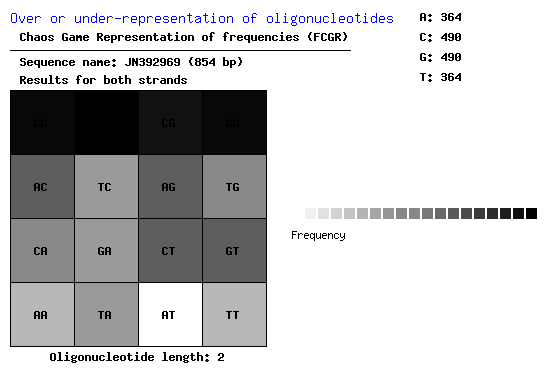


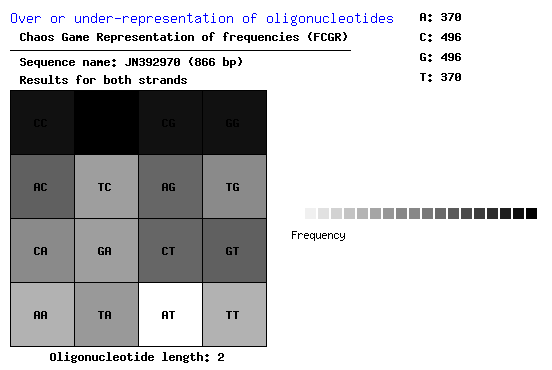


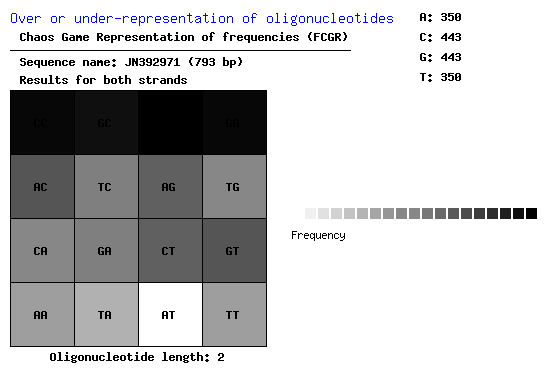


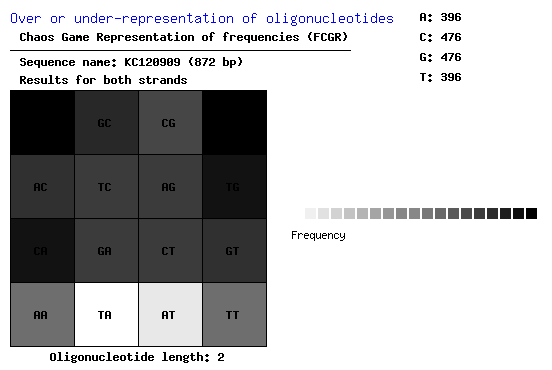

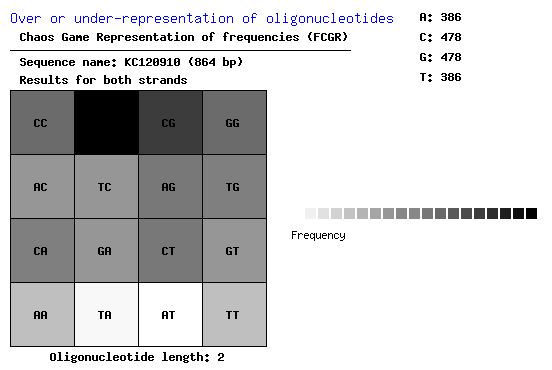


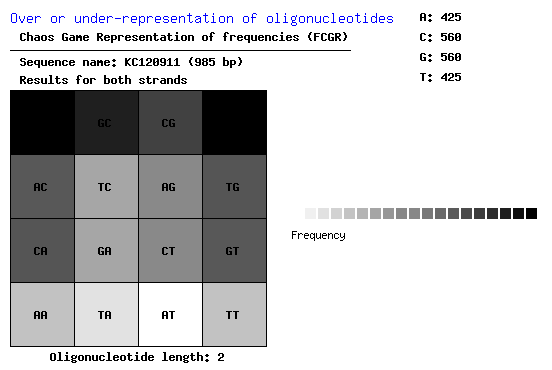


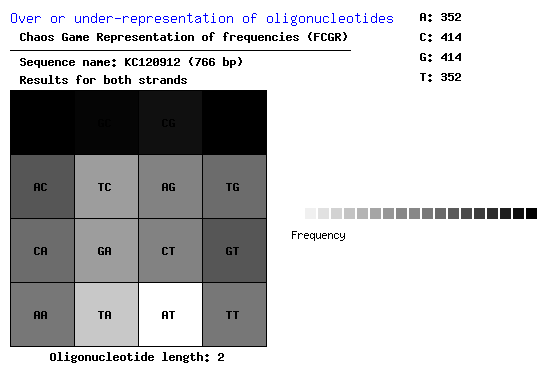


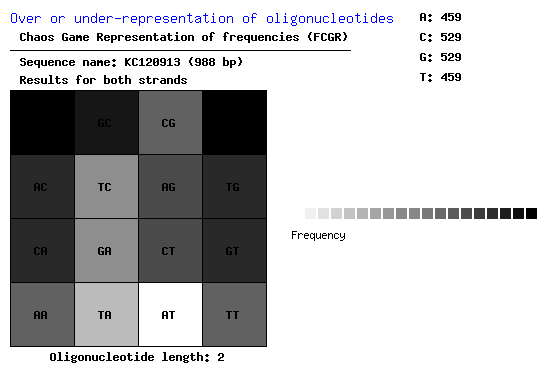


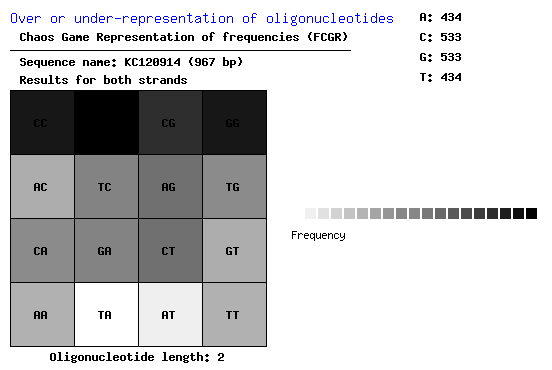


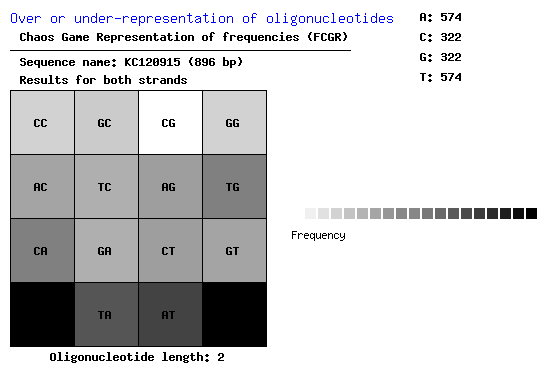


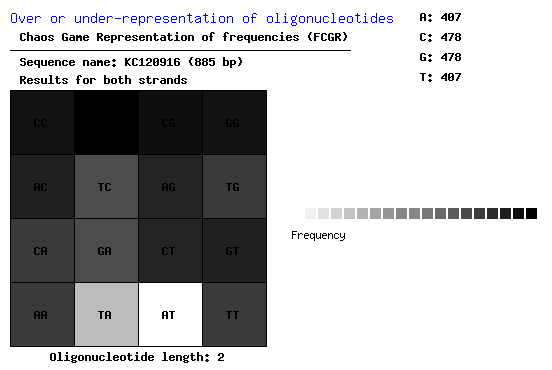


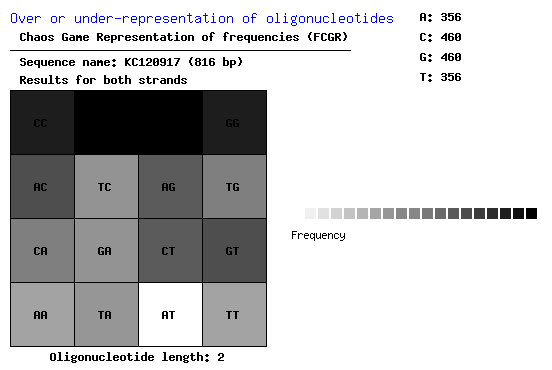


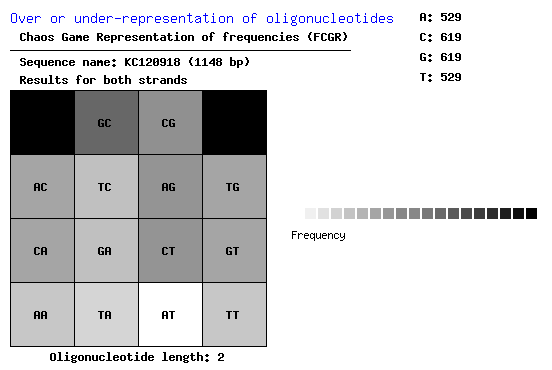


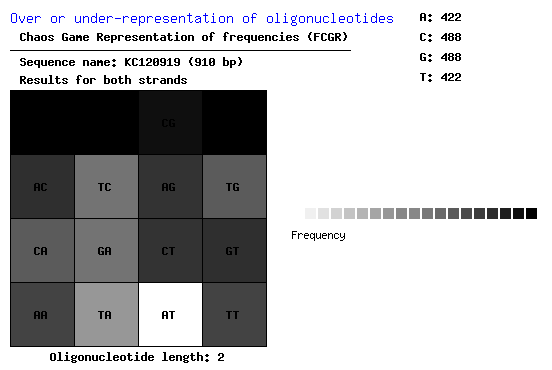


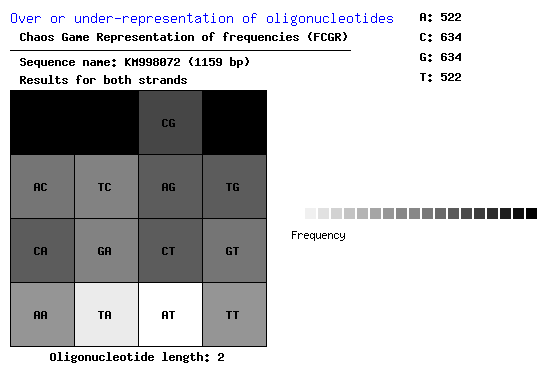


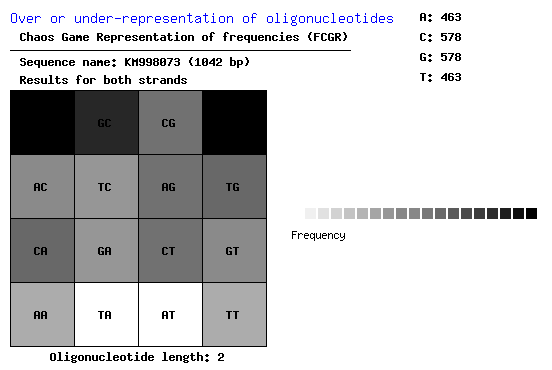


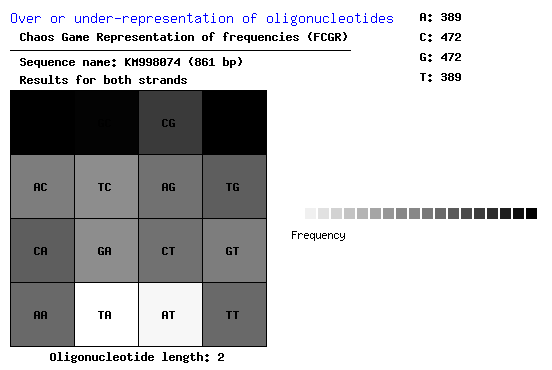


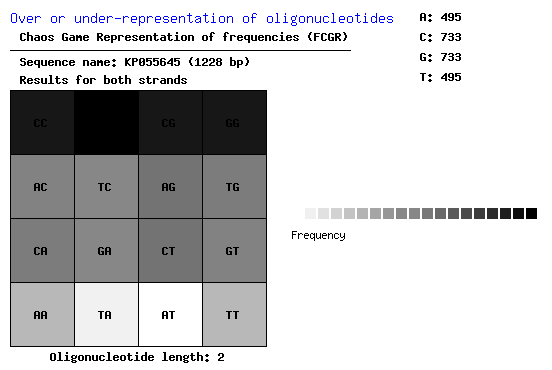


Figure: FCGR code of isolates

Supplement: Supplementary file 3 — Supplementary material [file mmc3.zip › Chaose Game Representation of Frequencies (FCGR)/fcgr.docx]

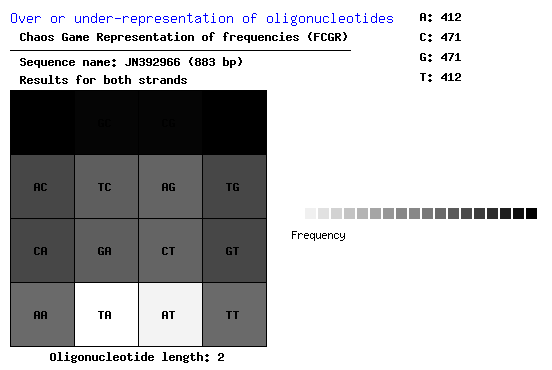

Supplement: Supplementary file 3 — Supplementary material [file mmc3.zip › Chaose Game Representation of Frequencies (FCGR)/JN392966.png]

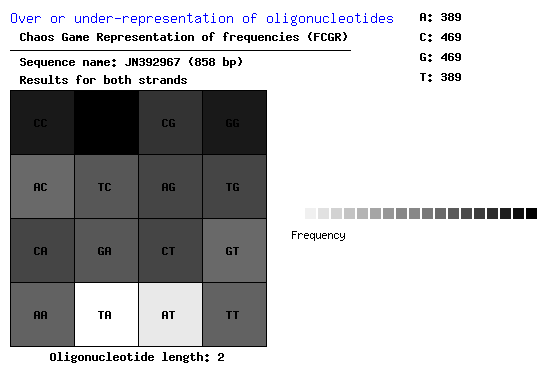

Supplement: Supplementary file 3 — Supplementary material [file mmc3.zip › Chaose Game Representation of Frequencies (FCGR)/JN392967.png]

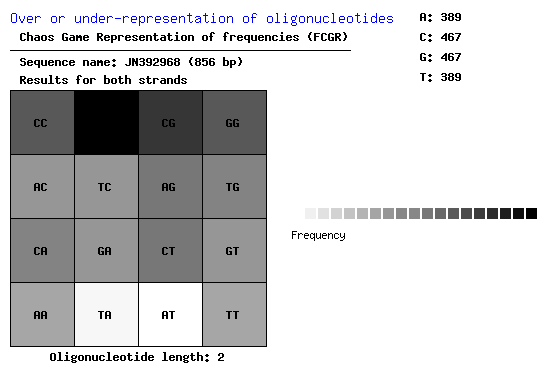

Supplement: Supplementary file 3 — Supplementary material [file mmc3.zip › Chaose Game Representation of Frequencies (FCGR)/JN392968.png]

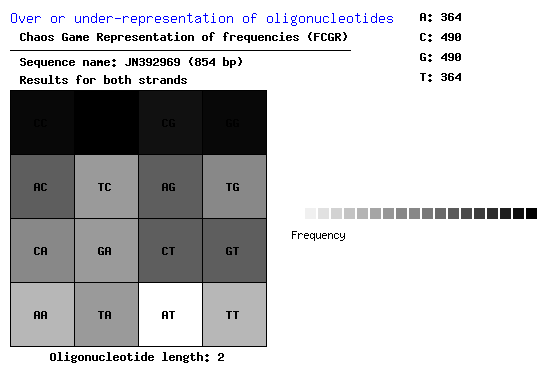

Supplement: Supplementary file 3 — Supplementary material [file mmc3.zip › Chaose Game Representation of Frequencies (FCGR)/JN392969.png]

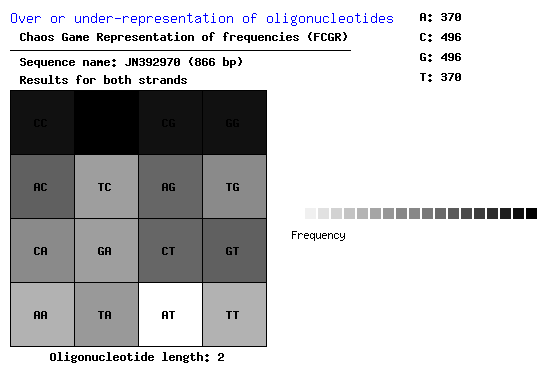

Supplement: Supplementary file 3 — Supplementary material [file mmc3.zip › Chaose Game Representation of Frequencies (FCGR)/JN392970.png]

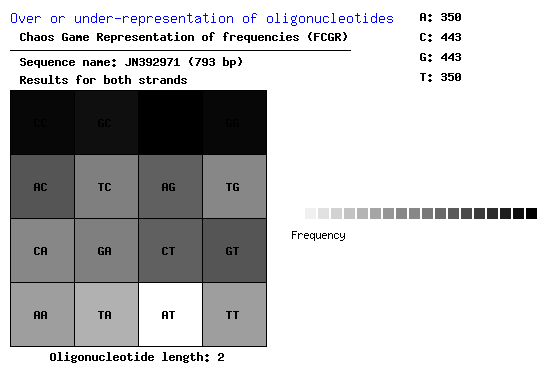

Supplement: Supplementary file 3 — Supplementary material [file mmc3.zip › Chaose Game Representation of Frequencies (FCGR)/JN392971.png]

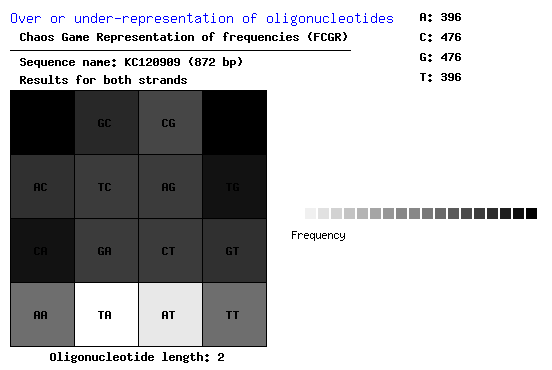

Supplement: Supplementary file 3 — Supplementary material [file mmc3.zip › Chaose Game Representation of Frequencies (FCGR)/KC120909.png]

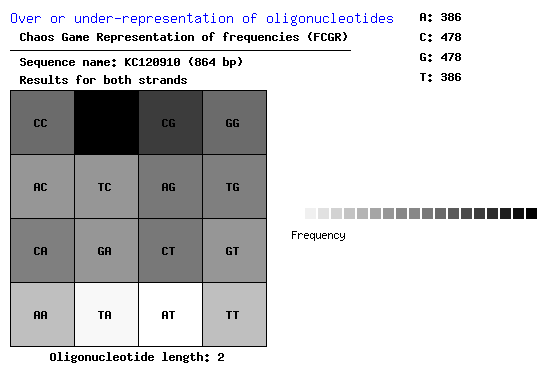

Supplement: Supplementary file 3 — Supplementary material [file mmc3.zip › Chaose Game Representation of Frequencies (FCGR)/KC120910.png]

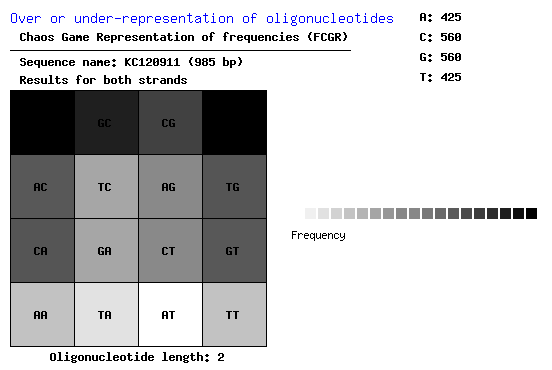

Supplement: Supplementary file 3 — Supplementary material [file mmc3.zip › Chaose Game Representation of Frequencies (FCGR)/KC120911.png]

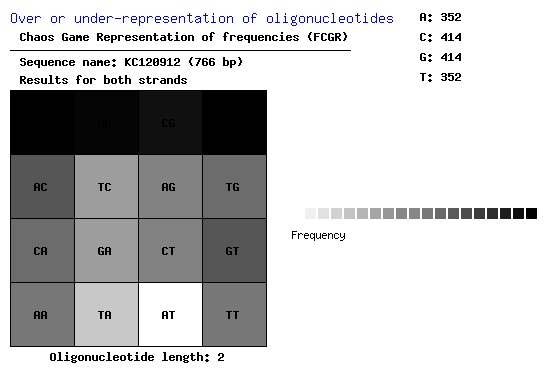

Supplement: Supplementary file 3 — Supplementary material [file mmc3.zip › Chaose Game Representation of Frequencies (FCGR)/KC120912.png]

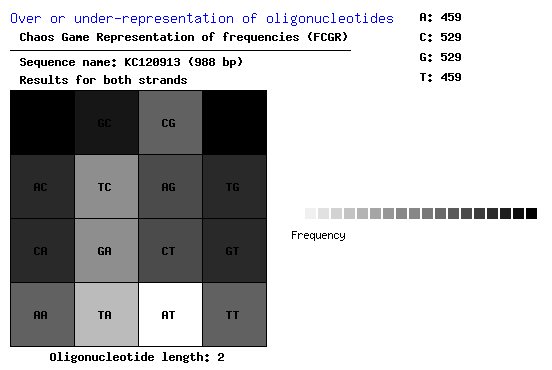

Supplement: Supplementary file 3 — Supplementary material [file mmc3.zip › Chaose Game Representation of Frequencies (FCGR)/KC120913.png]

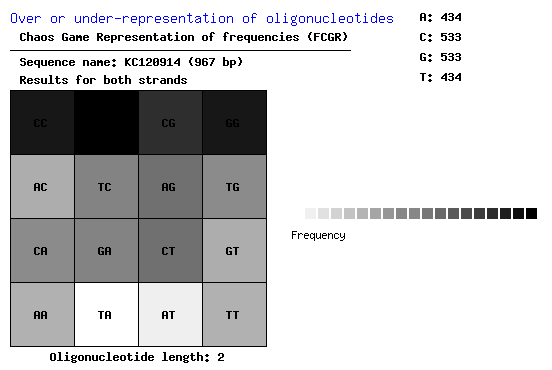

Supplement: Supplementary file 3 — Supplementary material [file mmc3.zip › Chaose Game Representation of Frequencies (FCGR)/KC120914.png]

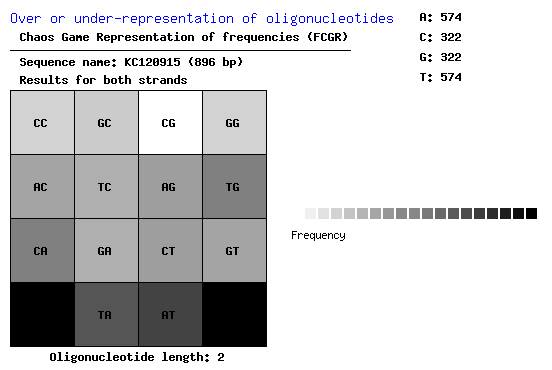

Supplement: Supplementary file 3 — Supplementary material [file mmc3.zip › Chaose Game Representation of Frequencies (FCGR)/KC120915.png]

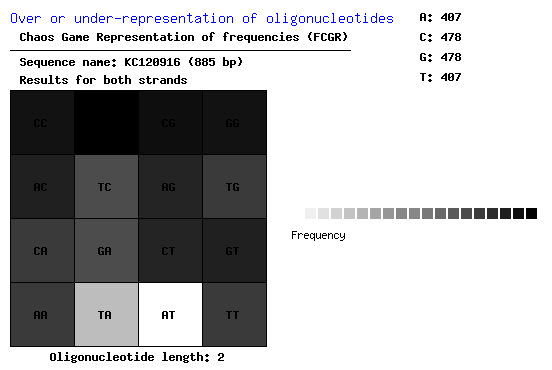

Supplement: Supplementary file 3 — Supplementary material [file mmc3.zip › Chaose Game Representation of Frequencies (FCGR)/KC120916.png]

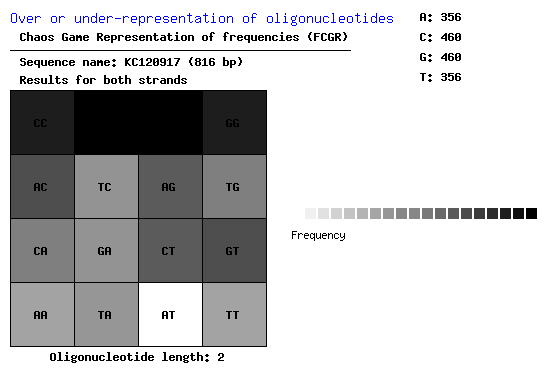

Supplement: Supplementary file 3 — Supplementary material [file mmc3.zip › Chaose Game Representation of Frequencies (FCGR)/KC120917.png]

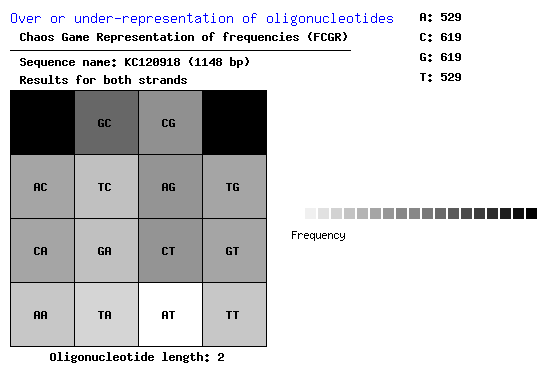

Supplement: Supplementary file 3 — Supplementary material [file mmc3.zip › Chaose Game Representation of Frequencies (FCGR)/KC120918.png]

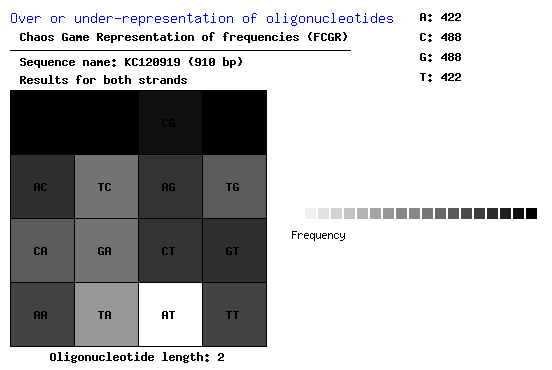

Supplement: Supplementary file 3 — Supplementary material [file mmc3.zip › Chaose Game Representation of Frequencies (FCGR)/KC120919.png]

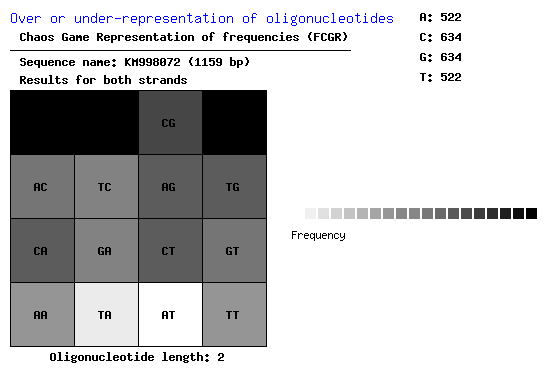

Supplement: Supplementary file 3 — Supplementary material [file mmc3.zip › Chaose Game Representation of Frequencies (FCGR)/KM998072.png]

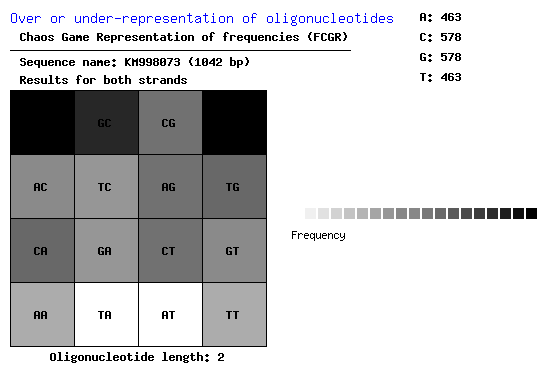

Supplement: Supplementary file 3 — Supplementary material [file mmc3.zip › Chaose Game Representation of Frequencies (FCGR)/KM998073.png]

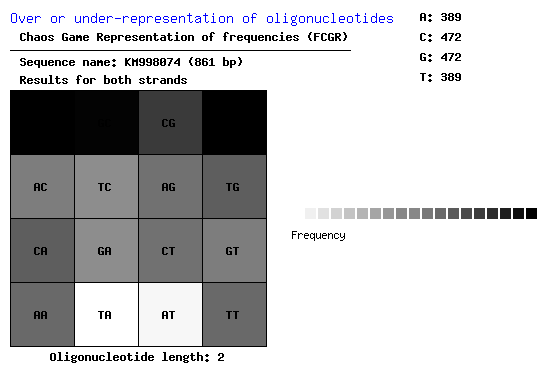

Supplement: Supplementary file 3 — Supplementary material [file mmc3.zip › Chaose Game Representation of Frequencies (FCGR)/KM998074.png]

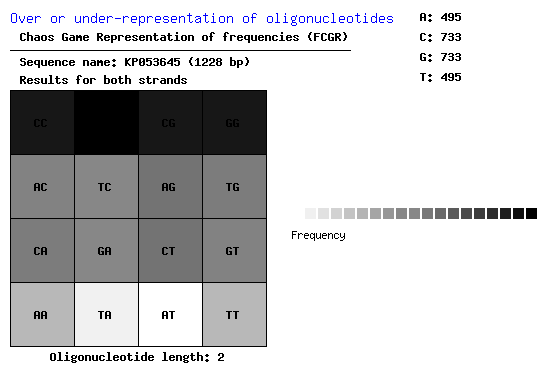

Supplement: Supplementary file 3 — Supplementary material [file mmc3.zip › Chaose Game Representation of Frequencies (FCGR)/KP053645.png]

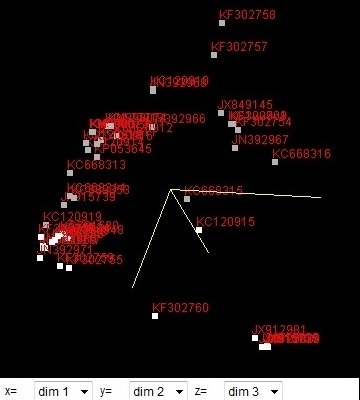

Supplement: Supplementary file 6 — Supplementary material [file mmc6.zip › Principal Componant Analysis (PCA) of 21 Thermophiles.jpg]

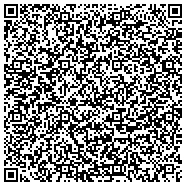

Supplement: Supplementary file 8 — Supplementary material [file mmc8.zip › JN392966.jpg]

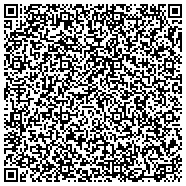

Supplement: Supplementary file 8 — Supplementary material [file mmc8.zip › JN392967.jpg]

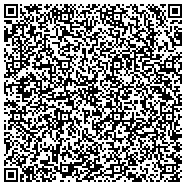

Supplement: Supplementary file 8 — Supplementary material [file mmc8.zip › JN392968.jpg]

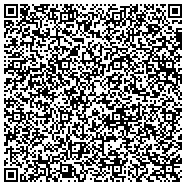

Supplement: Supplementary file 8 — Supplementary material [file mmc8.zip › JN392969.jpg]

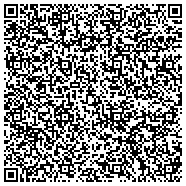

Supplement: Supplementary file 8 — Supplementary material [file mmc8.zip › JN392970.jpg]

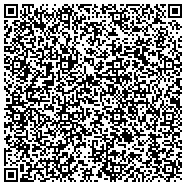

Supplement: Supplementary file 8 — Supplementary material [file mmc8.zip › JN392971.jpg]

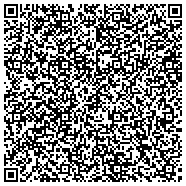

Supplement: Supplementary file 8 — Supplementary material [file mmc8.zip › KC120909.jpg]

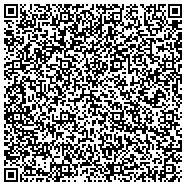

Supplement: Supplementary file 8 — Supplementary material [file mmc8.zip › KC120910.jpg]

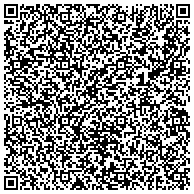

Supplement: Supplementary file 8 — Supplementary material [file mmc8.zip › KC120911.jpg]

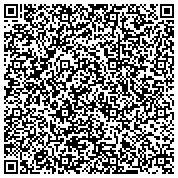

Supplement: Supplementary file 8 — Supplementary material [file mmc8.zip › KC120912.jpg]

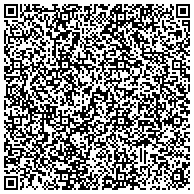

Supplement: Supplementary file 8 — Supplementary material [file mmc8.zip › KC120913.jpg]

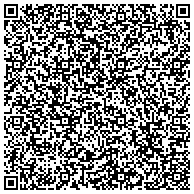

Supplement: Supplementary file 8 — Supplementary material [file mmc8.zip › KC120914.jpg]

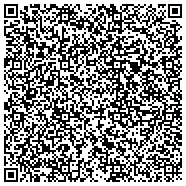

Supplement: Supplementary file 8 — Supplementary material [file mmc8.zip › KC120915.jpg]

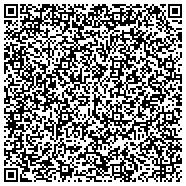

Supplement: Supplementary file 8 — Supplementary material [file mmc8.zip › KC120916.jpg]

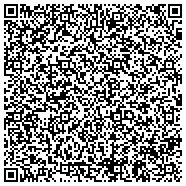

Supplement: Supplementary file 8 — Supplementary material [file mmc8.zip › KC120917.jpg]

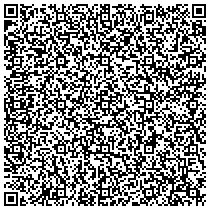

Supplement: Supplementary file 8 — Supplementary material [file mmc8.zip › KC120918.jpg]

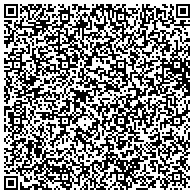

Supplement: Supplementary file 8 — Supplementary material [file mmc8.zip › KC120919.jpg]

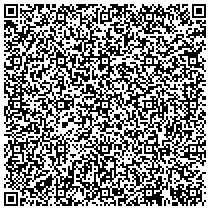

Supplement: Supplementary file 8 — Supplementary material [file mmc8.zip › KM998072.jpg]

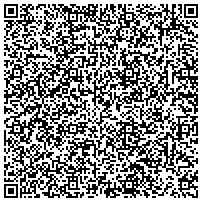

Supplement: Supplementary file 8 — Supplementary material [file mmc8.zip › KM998073.jpg]

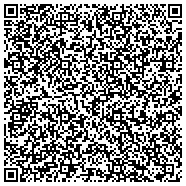

Supplement: Supplementary file 8 — Supplementary material [file mmc8.zip › KM998074.jpg]

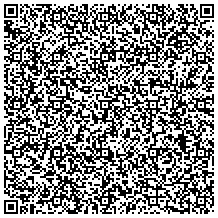

Supplement: Supplementary file 8 — Supplementary material [file mmc8.zip › KP053645.jpg]

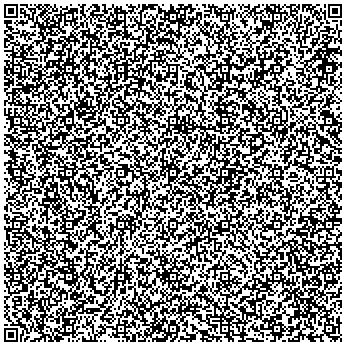

Supplement: Supplementary file 9 — Supplementary material [file mmc9.zip › JN392970.jpg]

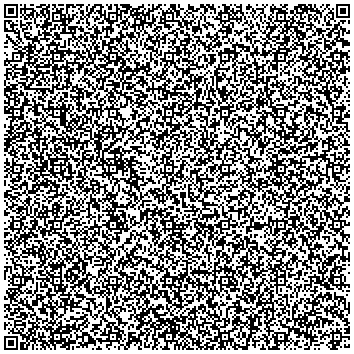

Supplement: Supplementary file 9 — Supplementary material [file mmc9.zip › JN392971.jpg]

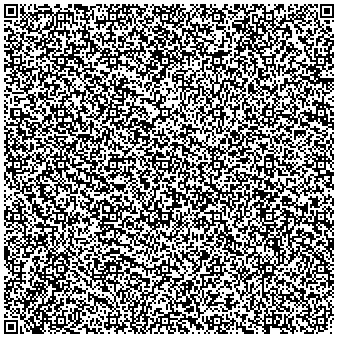

Supplement: Supplementary file 9 — Supplementary material [file mmc9.zip › KC120909.jpg]

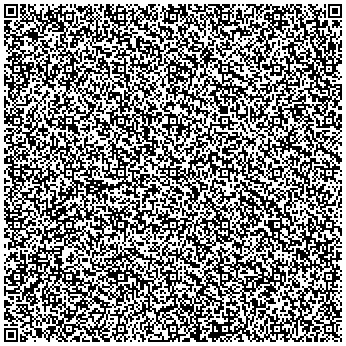

Supplement: Supplementary file 9 — Supplementary material [file mmc9.zip › KC120910.jpg]

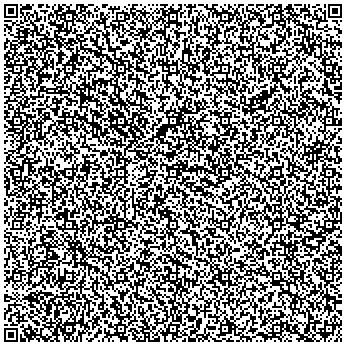

Supplement: Supplementary file 9 — Supplementary material [file mmc9.zip › KC120911.jpg]

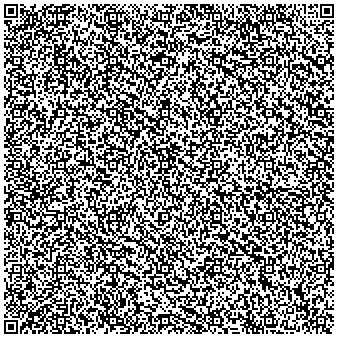

Supplement: Supplementary file 9 — Supplementary material [file mmc9.zip › KC120912.jpg]

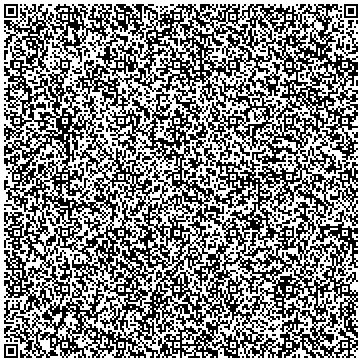

Supplement: Supplementary file 9 — Supplementary material [file mmc9.zip › KC120913.jpg]

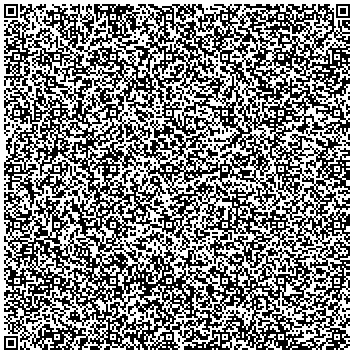

Supplement: Supplementary file 9 — Supplementary material [file mmc9.zip › KC120914.jpg]

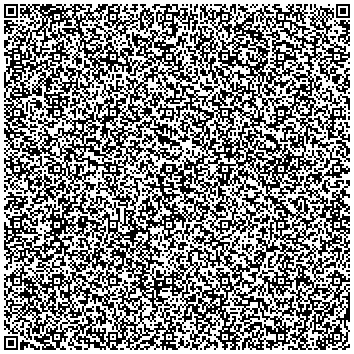

Supplement: Supplementary file 9 — Supplementary material [file mmc9.zip › KC120915.jpg]

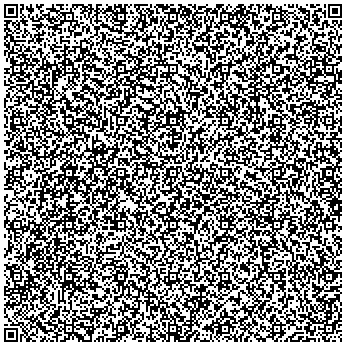

Supplement: Supplementary file 9 — Supplementary material [file mmc9.zip › KC120916.jpg]

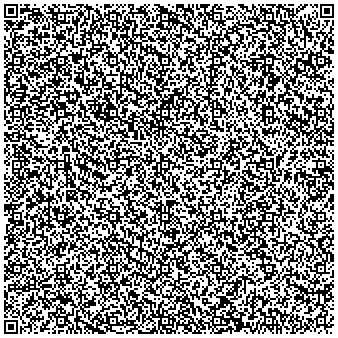

Supplement: Supplementary file 9 — Supplementary material [file mmc9.zip › KC120917.jpg]

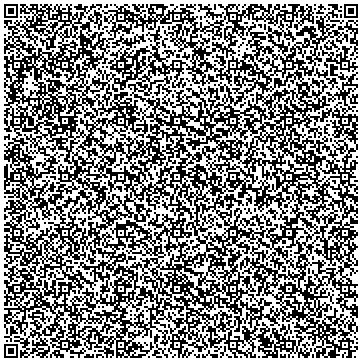

Supplement: Supplementary file 9 — Supplementary material [file mmc9.zip › KC120918.jpg]

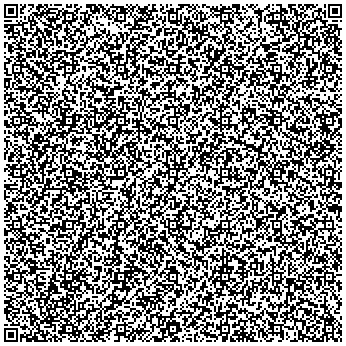

Supplement: Supplementary file 9 — Supplementary material [file mmc9.zip › KC120919.jpg]

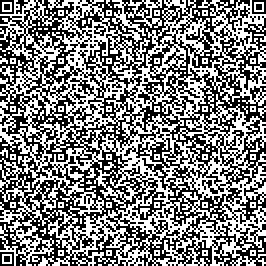

Supplement: Supplementary file 9 — Supplementary material [file mmc9.zip › KM998072.png]

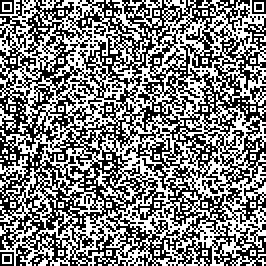

Supplement: Supplementary file 9 — Supplementary material [file mmc9.zip › KM998073.png]

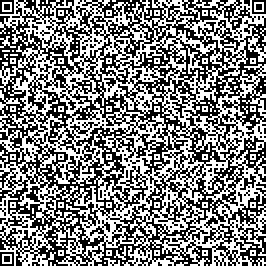

Supplement: Supplementary file 9 — Supplementary material [file mmc9.zip › KM998074.png]

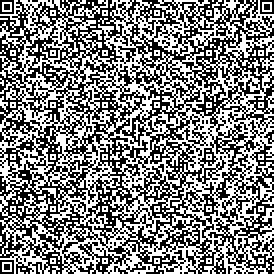

Supplement: Supplementary file 9 — Supplementary material [file mmc9.zip › KP053645.png]

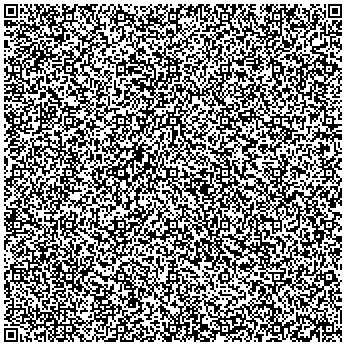

Supplement: Supplementary file 9 — Supplementary material [file mmc9.zip › JN392966.jpg]

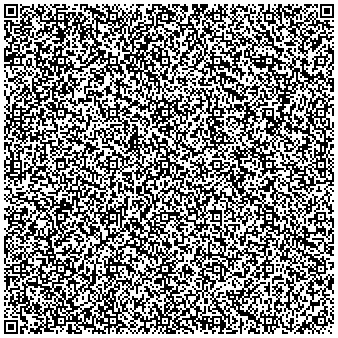

Supplement: Supplementary file 9 — Supplementary material [file mmc9.zip › JN392967.jpg]

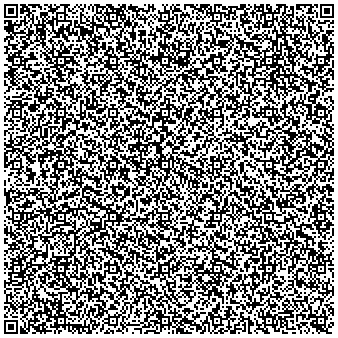

Supplement: Supplementary file 9 — Supplementary material [file mmc9.zip › JN392968.jpg]

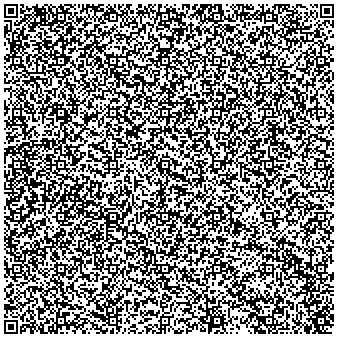

Supplement: Supplementary file 9 — Supplementary material [file mmc9.zip › JN392969.jpg]

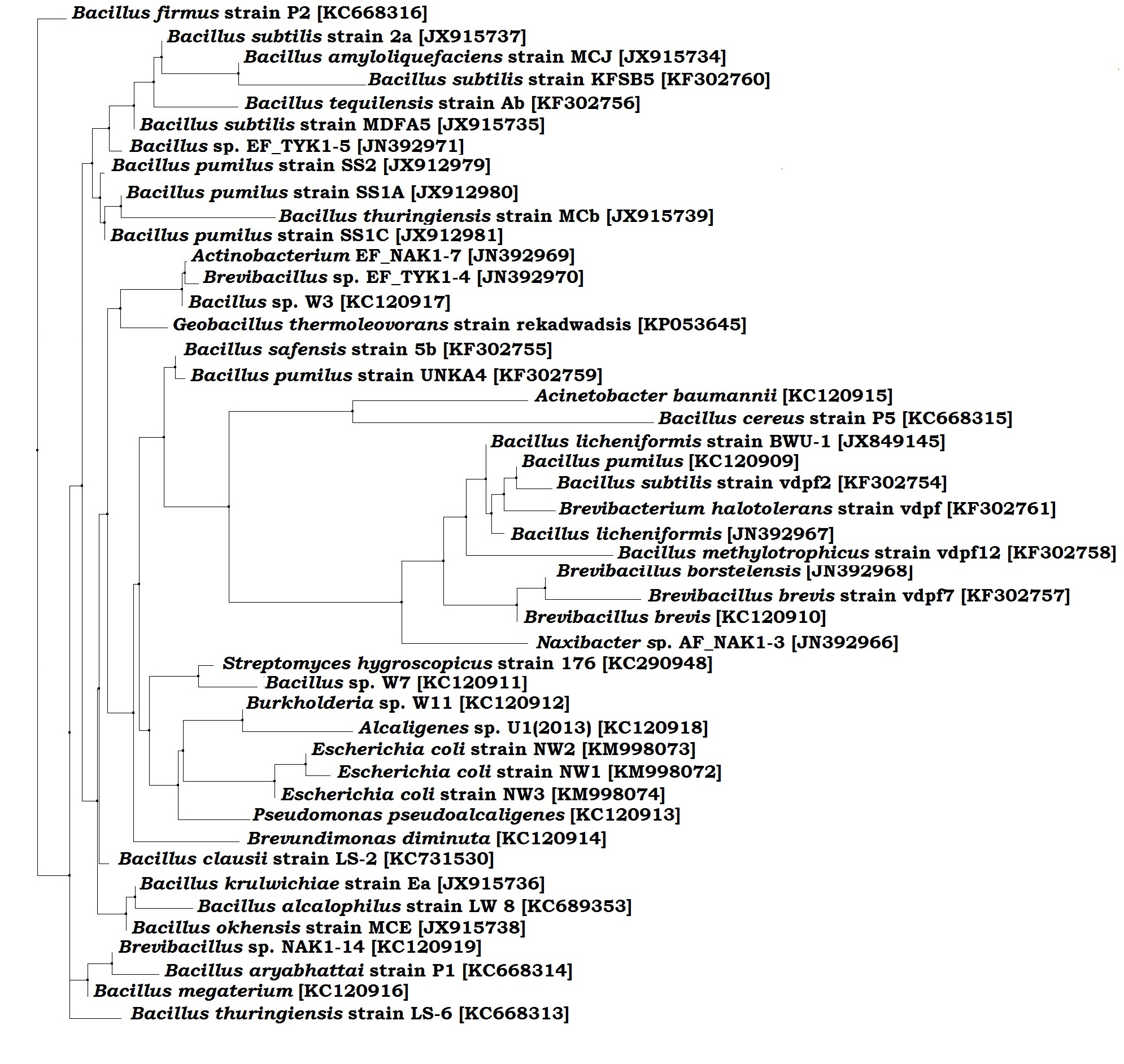

Supplement: Supplementary file 9 — Supplementary material [file mmc9.zip › Fig. 2 Neighbor joining plot of isolates JN392966-JN392971, KC120909-KC120919, KM998072-KM998074 and KP053645 constructed (using MEGA 6.02) with identified isolated from other hot springs..jpg]

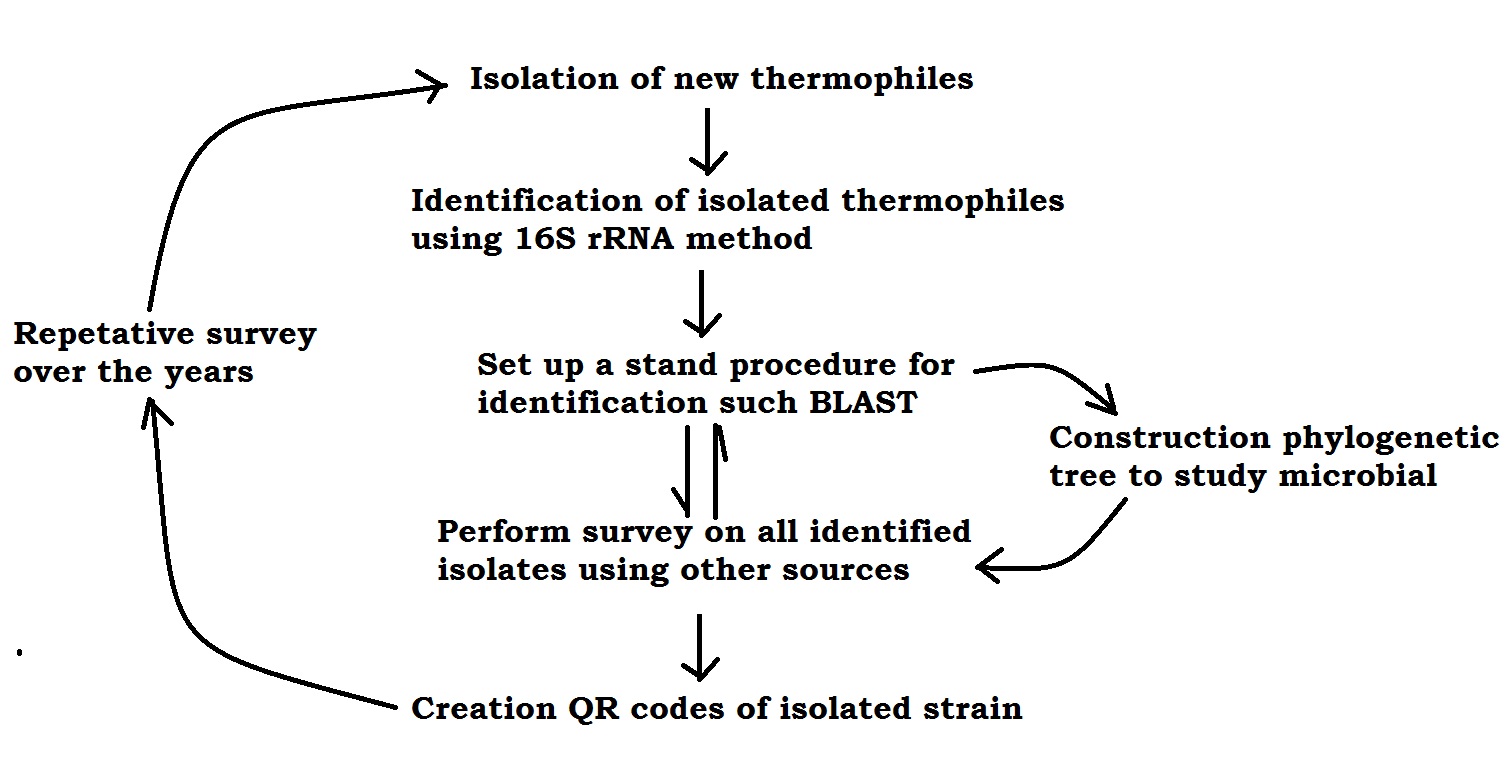

Supplement: Supplementary file 9 — Supplementary material [file mmc9.zip › Fig. 1 The diagram shows constructive step-by-step procedure to assess microbial diversity identification and creation of QR codes.jpg]
